# Targeted deep sequencing revealed variants in cell-free DNA of hormone receptor-positive metastatic breast cancer patients

**DOI:** 10.1007/s00018-019-03189-z

**Published:** 2019-06-28

**Authors:** Corinna Keup, Karim Benyaa, Siegfried Hauch, Markus Sprenger-Haussels, Mitra Tewes, Pawel Mach, Ann-Kathrin Bittner, Rainer Kimmig, Peter Hahn, Sabine Kasimir-Bauer

**Affiliations:** 1 Department of Gynecology and Obstetrics, University Hospital of Essen, Essen, Germany; 2 QIAGEN GmbH, Hilden, Germany; 3 Department of Medical Oncology, University Hospital of Essen, Essen, Germany

**Keywords:** Metastatic breast cancer, Liquid biopsy, Therapy stratification, Next generation sequencing, NGS, Circulating tumor DNA, ctDNA, ccfDNA, Mutation, SNP, Unique molecular indices

## Abstract

Cell-free DNA (cfDNA) is described to mirror intratumoral heterogeneity and gives insight about clonal evolution for improved therapeutic decisions. We sequenced cfDNA of a hormone receptor-positive, HER2-negative metastatic breast cancer (MBC) cohort with a high coverage to examine the prevalence and relevance of any detected variant. cfDNA of 44 MBC patients was isolated, followed by library construction using a customized targeted DNA panel with integrated unique molecular indices analyzing *AKT1*, *AR*, *BRCA1*, *BRCA2*, *EGFR*, *ERCC4*, *ERBB2*, *ERBB3*, *ESR1*, *FGFR1*, *KRAS*, *MUC16*, *PIK3CA*, *PIK3R1*, *PTEN*, *PTGFR,* and *TGFB1*. CfDNA was sequenced on the NextSeq^®^ 550 platform (Illumina) and variants were analyzed with Ingenuity Variant Analysis (QIAGEN). We evaluated cfDNA variants in 40 of the 44 hormone receptor-positive and HER2-negative patients with a high mean coverage of 22,000×, resulting in *MUC16*, *BRCA2*, *ERBB3,* and *AR* variant calling in > 90% of the patients. 47% of all *AR* variants were pathogenic and at least one pathogenic or likely pathogenic variant was detected in each patient. A specific *BRCA1* variant and > 3.5 pathogenic variants significantly associated with a reduced survival after diagnosis of metastasis. Longitudinal monitoring revealed an increase of pathogenic and likely pathogenic *PIK3CA* and *ESR1* variant allele frequency under everolimus and exemestane, 8 months before proof of therapy failure by visual staging in one exemplary case. The identification of new variants with high prevalence, prognostic value, and dynamics under treatment by deep sequencing of cfDNA might empower sensitive monitoring and personalized therapeutic decisions.

**Electronic supplementary material:**

The online version of this article (10.1007/s00018-019-03189-z) contains supplementary material, which is available to authorized users.

## Introduction

Currently, liquid biopsies appear as promising tools for individualized treatment decisions and real-time monitoring strategies in oncology including breast cancer (BC). Cell-free DNA (cfDNA), specifically cell-free tumor DNA (ctDNA), defined by the presence of variants [1], represents tumor heterogeneity, because ctDNA can be released by tumor cells in the primary tissue, in metastases, and in circulation. Moreover, ctDNA harbors newly acquired variants selected by and/or expanding under therapy [2], therefore, mirroring clonal evolution. In contrast, tissue biopsies only depict a spatially and temporally limited snapshot and are often not feasible. CfDNA, reflecting the characteristics of the metastases better than those of the primary tumor [3], could serve as easily assessable alternative for biopsies of metastases.

The relevance of cfDNA in BC has already been reported for diagnostic, prognostic, predictive, and monitoring approaches [4]. The prognostic value of ctDNA is undisputable in BC, since high levels of ctDNA were associated with poor overall survival (OS) [5]. *ESR1* variants were specifically correlated with shorter duration of endocrine treatment effectiveness in metastatic BC (MBC) [6] and *PIK3CA* variants in exon 20, significantly associated with poor prognosis [7]. Regarding the predictive value, patients with *ESR1* variants were demonstrated to benefit from fulvestrant rather than from exemestane, compared to patients without this somatic alteration [8]. In the BELLE-2 study, the addition of a pan-PI3K inhibitor resulted in an improved progression-free survival only in patients with *PIK3CA*-altered cfDNA [9]. For disease monitoring, cfDNA concentration was reported to indicate impending relapse of primary BC earlier than any other imaging or blood-based strategy [10] and could predate treatment response changes [11, 12].

Detection methods for cfDNA variants are as diverse as their advantages and disadvantages [1, 13]. The rapid development of next generation sequencing (NGS) methods supported the unbiased variant analysis in the cfDNA research field. Due to the low input requirements, targeted PCR-based library techniques are preferred over hybrid-capture methods [14]. However, PCR-based techniques are prone to artifacts due to the error rate of polymerases and, thus, display a decreased sensitivity of around 1% [1]. Modern molecular barcoding techniques handle this problem and bioinformatically remove PCR artifacts to detect true positive variants at allele frequencies (AFs) of down to 0.1% [4, 10].

We here (a) established a targeted PCR-based NGS approach with integrated unique molecular indices (UMIs) and extremely high coverage to analyze cfDNA variants from hormone receptor (HR)**-**positive and HER2**-**negative MBC patients in detail and (b) compared results with and without UMIs, (c) examined variants in *MUC16*, *BRCA2*, *ERBB3*, AR, *EGFR*, *PTEN*, *ERBB2*, *PIK3CA*, *BRCA1*, *ESR1*, *PTGFR*, *TGFB1*, *AKT1*, *FGFR1*, *ERCC4*, *PIK3R1,* and *KRAS,* and (d) explored the development of likely pathogenic and pathogenic cfDNA variants of one particular HR+ HER2**–** patient from the time point of primary tumor biopsy until death to get insight about the value of cfDNA variants for treatment decision making and monitoring.

## Subjects and methods

### Patient population characteristics and eligibility criteria

The study was carried out at the Department of Gynecology and Obstetrics, in collaboration with the Department of Medical Oncology (for specimen recruitment), both at the University Hospital Essen, Germany and in collaboration with QIAGEN GmbH, Hilden, Germany (for library preparation and sequencing analysis). The eligibility criteria have been previously published [15]. Written informed consent was obtained from all participants at enrollment and specimens were collected using protocols approved by the institutional review board (12-5265-BO). In total, cfDNA from 44 MBC patients was studied between March 2013 and August 2017. MBC patients with estrogen (ER) and/or progesterone (PR) receptor-positive primary tumors without *ERBB2* overamplification were enrolled. Patients with ER**-**positive and/or PR**-**positive and HER2**-**negative metastases (if multiple pathology reports of metastases were available, we used the report of metastasis biopsy that was taken closest to the date of blood draw for this study) had also been included if their ER, PR and HER2 status in the primary tumor was unknown. Patient characteristics of the 40/44 cases finally fulfilling the stringent sequencing quality parameter and thus used for variant analysis are listed in Online Resource 1.

### Sampling of blood, processing of plasma, and isolation of cfDNA

9 ml EDTA blood was collected in S-Monovettes^®^ (Sarstedt, Germany), stored at 4 °C, and centrifuged within 4 h after withdrawal at 1 841 × g for 8 min. Plasma was stored at − 80 °C. Thawed plasma was centrifuged at 16,000×*g* for 10 min at 4 °C and passed through a 0.8 µm pore size syringe filter (Sartorius, Germany). cfDNA was isolated from 1.8 to 5.4 ml (preferably 4 ml) plasma by affinity-based binding to magnetic beads according to the manufacturer’s instructions (QIAamp MinElute ccfDNA Kit, QIAGEN, Germany) and was eluted in 22 µl ultraclean water.

### cfDNA quantification

Diluted cfDNA (1:2–1:100) was applied to the Agilent Chip High Sensitivity DNA (Santa Clara, US). Concentrations of fragments with a length between 100 and 700 bp were added up using the 2100 expert software B02.08 to calculate the cfDNA yield.

### Library construction

The library was constructed with the QIAseq Targeted DNA Panel Kit (QIAGEN) and according the manufacturer’s instructions. The input amount preferred for library preparation was in the range of 30–60 ng, but cfDNA samples with lower input were also included in the library preparation. Because the concentration of some cfDNA eluates was critically low, we used an input volume of 20 µl for all libraries, instead of 16.75 µl as described in the handbook. Thus, the reagent volumes had to be adjusted as described and published at protocols.io [16]. Briefly, end-repair and a-addition was performed, while the enzymatic fragmentation was inhibited. In the next step, the UMI and sample-specific adapter were ligated to the fragments [14, 17]. DNA was purified and size selected by magnetic beads. The targeted enrichment was performed with customized QIAGEN QIAseq Targeted DNA Panel primer designed to amplify all coding regions of *AKT1*, AR, *BRCA1*, *BRCA2*, *EGFR*, *ERBB2*, *ERBB3*, *ERCC4*, *ESR1*, *KRAS*, *FGFR1*, *MUC16*, *PIK3CA*, *PIK3R1*, *PTEN*, *PTGFR*, and *TGFB1* and exhibited high specificity and uniformity (Online Resource 2). The universal PCR amplification and integrated additional sample-specific adapter ligation was followed by a magnetic bead cleanup and the final targeted enriched cfDNA library was eluted.

### Sequencing

Libraries were quantified as published at protocols.io [16] by qPCR and the quality was checked by Agilent Chip High Sensitivity DNA. Libraries were diluted to 2 nM (MiSeq, Illumina) or 4 nM (NextSeq, Illumina). Libraries with a lower yield were excluded. All pooled libraries were analyzed by in total four runs paired-end sequencing on two different Illumina sequencers using a custom sequencing primer (QIAseq A Read1 Primer). One run was performed on the Illumina MiSeq Sequencer with the MiSeq Reagent Kit v2, 2 × 150 bp reads with some samples for the comparison ± UMIs. All other libraries were sequenced on the Illumina NextSeq Sequencer with a NextSeq 550 System High-Output Kit, 2x150 bp reads.

### Data analysis/bioinformatical analysis

Data were initially analyzed using the QIAGEN GeneGlobe Data Analysis Center. Sufficient sequencing quality of all samples for clinical interpretation had been guaranteed by exclusion of libraries with less than 5 million read fragments, an UMI coverage lower than 400 and if less than 95% of the target region was covered with at least 5% of the mean UMI coverage, as described [16, 18]. The QIAGEN Biomedical Genomics Workbench and the Ingenuity Variant Analysis plugin (IVA; QIAGEN) were used for further annotation, scoring, filtering (described previously [16, 18]) and interpretation of variants detected in the UMI-based analysis. Called variants were separately annotated for the different transcript isoforms. Original raw sequencing data are available at the European Nucleotide Archive with the study accession number PRJEB29032 and sequencing quality parameter are listed in Online Resource 3.

### Statistical analysis

Cut-off values for subsequent survival analysis were determined by empirical ROC analysis and a maximized Youden’s index. Kaplan–Meier survival analysis was interpreted by log-rank (Mantel–Cox) test for all variants with a frequency of 15–85% in the cohort and for the parameters: number of variants detected, number of likely pathogenic and pathogenic variants detected, and mean AF of all detected variants. Significant values were indicated as asterisks (*≙*p* value < 0.05). Statistical analysis was performed by SPSS, version 11.5 (SPSS Inc.).

## Results

### Integration of UMIs in the library and its consequences on called *PIK3CA* hotspot variants

**Fig. 1:**
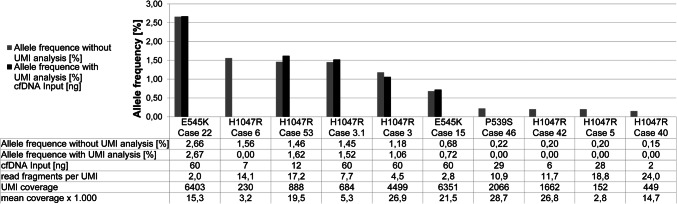
*Comparison of called PIK3CA hotspot variants with (black) and without (grey) unique molecular indices (UMIs).* Only cases with variant AFs < 3% are examined. Detection of low fraction *PIK3CA* hotspot variants with UMIs is dependent on high cfDNA input, as well as on a high UMI coverage and a small ratio of read fragments per UMI

To demonstrate the differences in results with and without use of UMIs, two different bioinformatical strategies were compared in samples that presented *PIK3CA* hotspot variants (P536S, E545K, and H1047R) with an AF < 3% in the workflow without UMI consolidation (*n* = 10). After integration of UMIs in the bioinformatical analysis 50% of the called variants without UMI consolidation were verified as true positives (Fig. 1). The *PIK3CA* hotspot variant with the lowest AF (0.72%) verified as true positive by UMIs was detected in a sample with a high cfDNA input (60 ng) and deep sequencing qualities (mean coverage: 22,000x, UMI coverage: 6351). In contrast, a sample with low cfDNA input (7 ng) sequenced on the MiSeq (mean coverage: 3,000x, UMI coverage: 230) failed in verification of *PIK3CA* H1047R detected without UMI consolidation (AF: 1.56%). In conclusion, high cfDNA input amount and corresponding good sequencing qualities enable verification of true positive variants by UMIs. Consequently, results described in the following sections were all confirmed by consolidation of UMIs.

### Insight into the characteristics of variants found in the HR+ HER2– MBC cohort

For the evaluation of clinically relevant insights disclosed in cfDNA, cfDNA was isolated of a histologically confirmed cohort of 44 HR+ HER2– MBCs. After sequencing, data of 4/44 samples were excluded due to application of stringent exclusion criteria for sequencing quality parameters. Sequencing quality parameter of the remaining 40 samples and corresponding patient characteristics are listed in the Online Resources 1 and 3. Importantly, a mean of 18 million fragment reads was analyzed in each sample with a mean coverage of 21,600× and a mean UMI coverage of 2668 indicating deep sequencing quality of all samples. Variants with a prevalence of > 3% in the normal reference population were excluded unless the variant was already known to be a pathogenic common variant.

**Fig. 2:**
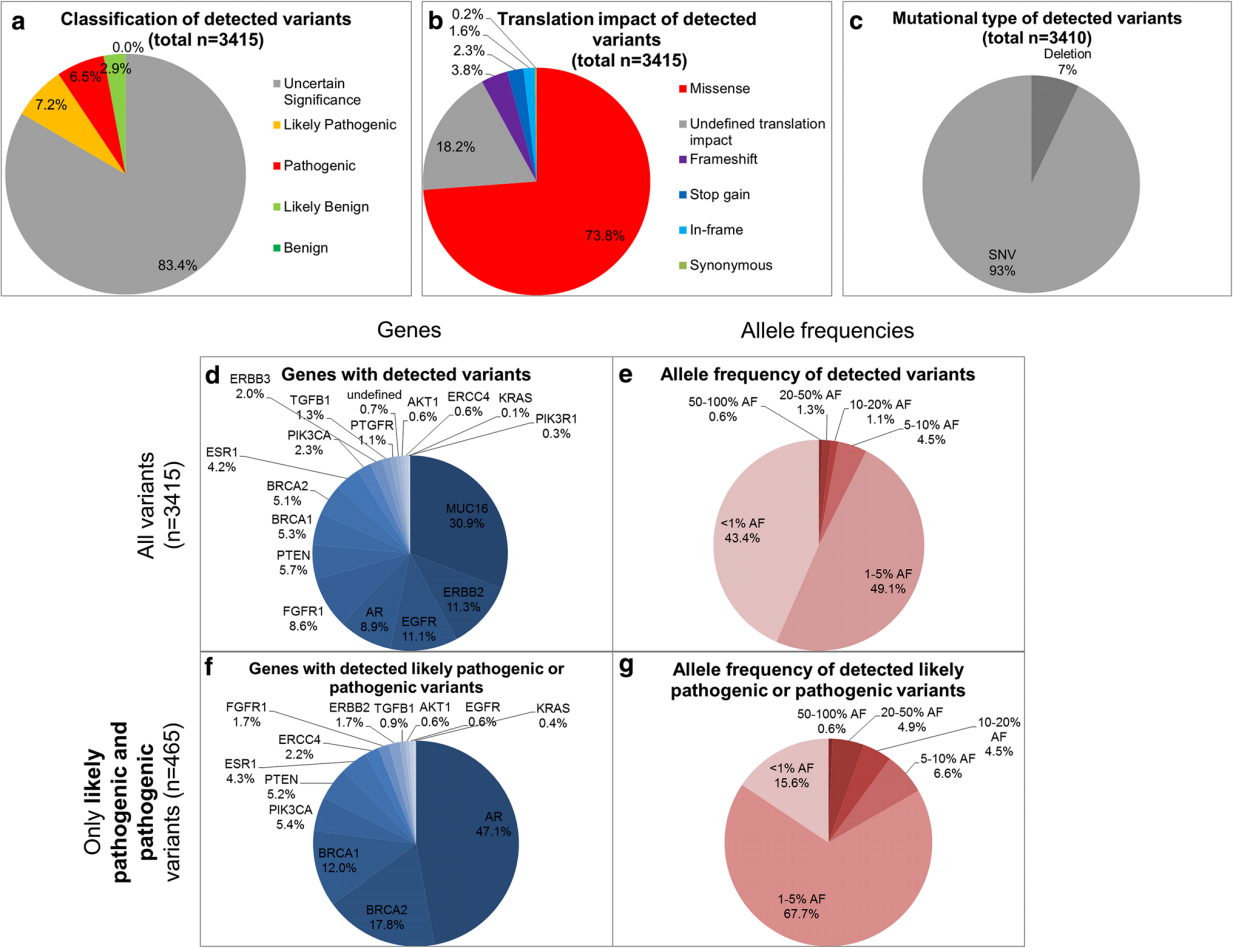
*Characteristics of called variants in cfDNA in HR+ HER2–* *MBC patients (n= 40).***a** Classification of variants according to their known impact (benign, likely benign, uncertain significance, likely pathogenic and pathogenic) [26] done by IVA. Of all detected variants (*n* = 3415), 14% are likely pathogenic or pathogenic. **b** Translation impact of detected variants. Three quarter of all variants were missense variants. **c** Differentiation into mutational types. Only *n* = 5 were multi nucleotide polymorphisms or insertions, while 93% were single nucleotide polymorphisms. **d** Distribution of variants regarding their gene location. Most variants were found in the *MUC16* gene. **e** AFs of all variants. 90% of all variants displayed AFs < 5%. **f** Distribution of all pathogenic or likely pathogenic variants (*n* = 465) regarding their gene location. Most pathogenic or likely pathogenic variants found in the *AR* gene. **g** AFs of all pathogenic and likely pathogenic variants. 68% presented an AF between 1 and 5%

In total, 3415 variants were found in the cfDNA of all patients sequenced using a targeted PCR-based NGS approach with integrated UMIs analyzing *MUC16*, *BRCA2*, *ERBB3*, *AR*, *EGFR*, *PTEN*, *ERBB2*, *PIK3CA*, *BRCA1*, *ESR1*, *PTGFR*, *TGFB1*, *AKT1*, *FGFR1*, *ERCC4*, *PIK3R1,* and *KRAS*. Of these detected variants, 6.5%/7.2% were identified as pathogenic/likely pathogenic by IVA, while 83.4% variants were of uncertain significance (Fig. 2a). Of all variants, 74% were missense variants (Fig. 2b) and 93% were single nucleotide polymorphisms (Fig. 2c).

Of all 3415 variants, 31% were found in the *MUC16* gene that thus was the gene with the most variants detected of all 17 tested genes (Fig. 2d). *ERBB2* was the gene with the second highest number of variants detected (11.3%). Interestingly, half of the detected variants (49%) showed an AF of between 1 and 5% and 43% of all variants were detected with an AF of < 1% (Fig. 2e).

Of all variants, 465/3415 variants were described to be pathogenic or likely pathogenic using IVA. Strikingly, 47% of all pathogenic or likely pathogenic variants were found in the *AR* gene (Fig. 2f); 5.4%/4.3% of all pathogenic and likely pathogenic variants were detected in the *PIK3CA*/*ESR1* gene. The fraction of variants with an AF of 20–50% increased by filtering only pathogenic and likely pathogenic variants from all variants (4.9% compared to 1.3%; Fig. 2e, g).

### Prominent variants in the HR+ HER2– MBC cohort

**Fig. 3:**
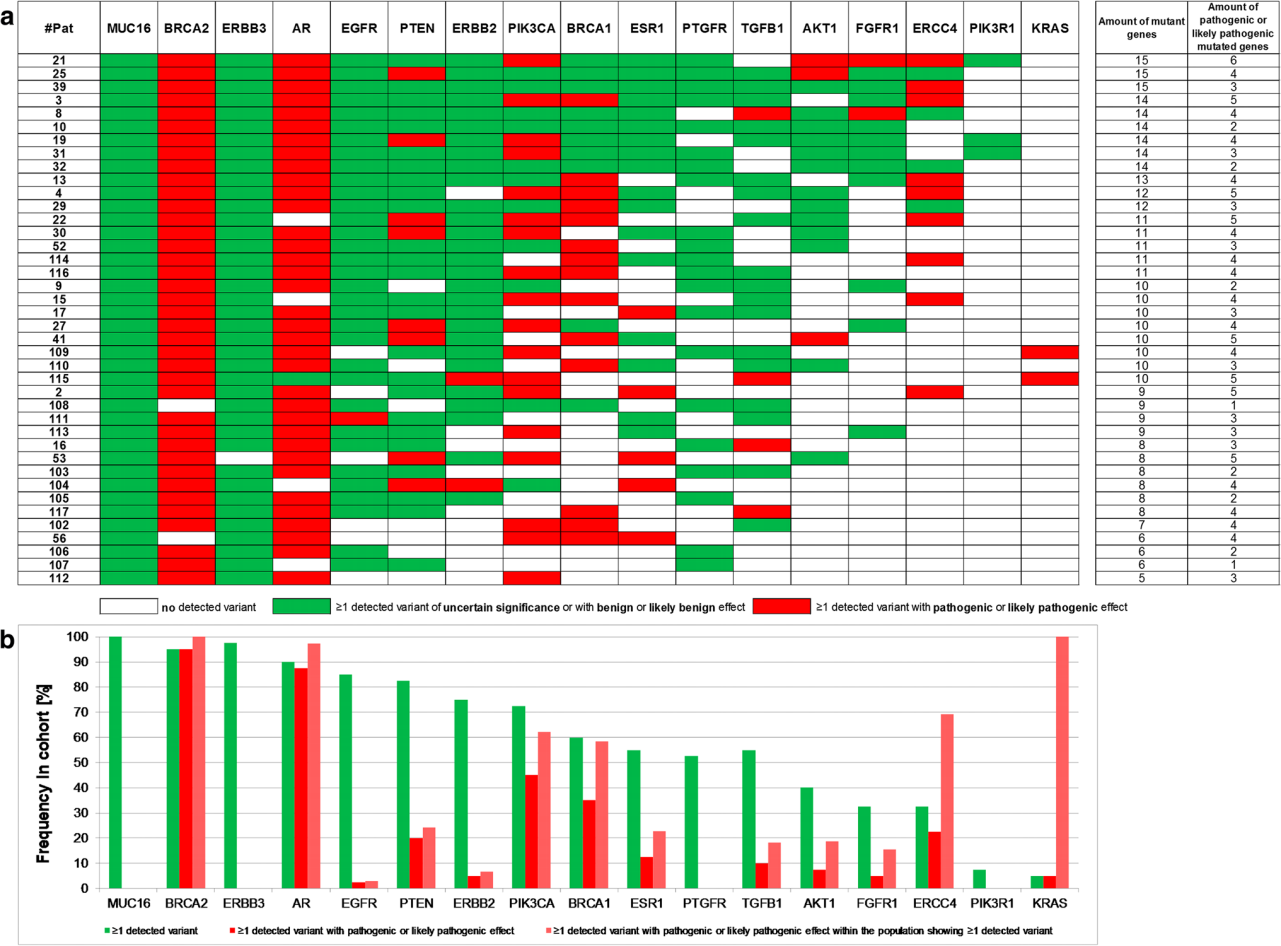
*Prevalence of altered genes.***a** Heatmap of mutated genes in each of the 40 patients. Genes with at least one variant are illustrated in color. Genes with at least one likely pathogenic or pathogenic variant are presented in red. Patients were sorted according to the amount of altered genes. Each patient displayed at least one pathogenic or likely pathogenic variant in one of the 17 tested genes and showed at least one *MUC16* variant. **b** Prevalence of altered genes in percentage. Frequency of altered genes (green), frequency of pathogenic or likely pathogenic altered genes (red) and frequency of pathogenic or likely pathogenic alterations within the cohort showing any variant in this gene (pink) are depicted. *MUC16*, *BRCA2*, *ERBB3* and *AR* alterations showed a prevalence of > 90%. *BRCA2* and *AR* were the most frequently altered genes with pathogenic or likely pathogenic effect

Breaking down the genetic localization of the detected variants for each of the 40 HR+ HER2**–** MBC patient (Fig. 3) revealed that each patient showed at least one gene with a likely pathogenic or pathogenic variant.

*MUC16* variants were found in each patient (100%) and the frequency of at least one variant in *BRCA2*, *ERBB3,* and *AR* was > 90% (Fig. 3). Except *KRAS* and *PIK3R1*, all tested genes displayed variants in at least 30% of all patients (for example, *PIK3CA* 73% and *ESR1* 55%).

Twelve *MUC16* variants, six *PTEN* variants, four *ERBB2*, *AR,* and *EGFR* variants, and two *BRCA2* variants were detected with a prevalence of > 50% (Online Resource File 4). The two most common variants in the cohort were *MUC16* c.39020T > G p.V13007G, detected in 38/40 patients (95% prevalence, mean AF 3.2%) and *MUC16* c.17434_17436delACT p.T5812del identified in 34/40 samples (85%; mean AF 0.8%) (Online Resource 4).

### Pathogenic and likely pathogenic variants in the HR+ HER2– MBC cohort

Interestingly, 95% and 88% of all patients displayed likely pathogenic or pathogenic variants in *BRCA2* and *AR* (Fig. 3).

All identified likely pathogenic or pathogenic variants and their corresponding AF in the respective patient samples are listed in Online Resource 5. The likely pathogenic variants or pathogenic *ESR1* variants mostly appeared as cluster of more than one *ESR1* variant with the same AFs within one patient. A diversity of likely pathogenic or pathogenic *PIK3CA* variants (K1111del, P539S, G912E, G1007R, R88Q, E542K, E545K, E545A, E726K, and H1047R) was detected. The highest prevalence of a pathogenic variants was found for *BRCA2* E1299* (78%) and *BRCA2* T3033fs*29 (75%). The exonic variant with likely pathogenic impact and greatest prevalence was *AR* H382P (80%).

### Prognostic value of cfDNA variants

The median survival time in the HR+ HER2**–** MBC cohort (*n* = 40; Online Resource 1) after diagnosis of metastasis was 46 months with an interquartile range (IQR) of 31 months. At the examination time point for the prognostic value of the detected variants in the cohort by Kaplan–Meier analysis, 29 patients were deceased, while 11 were still alive (Online Resource 1).

**Fig. 4:**
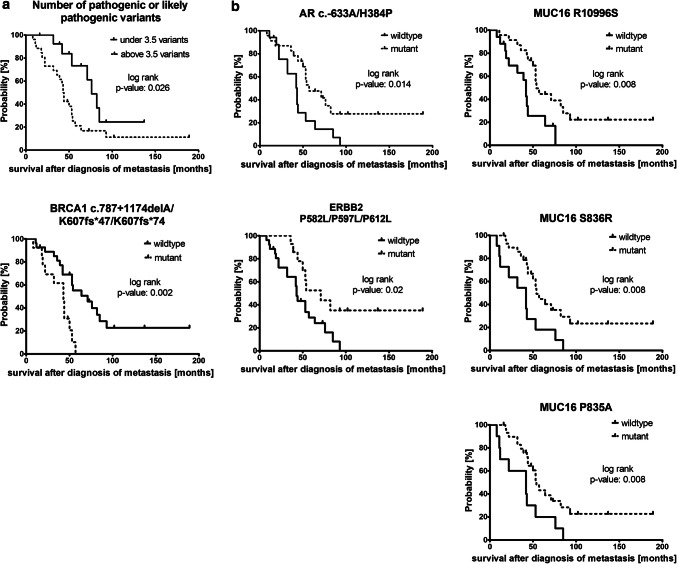
*Survival analysis by Kaplan–Meier curves and log-rank (Mantel–Cox) test.a* Kaplan–Meier curves illustrating parameter significantly correlated with reduced survival after diagnosis of metastasis. Cut-off values were calculated by ROC analysis and Youdens’ index. Patients with > 3.5 pathogenic and likely pathogenic variants or with *BRCA1* variant c.787 + 1174delA/K607fs*47/K607fs*74 (different annotations according to different *BRCA1* isoforms) displayed a significant correlation with worse survival after diagnosis of metastasis. **b** Single variants significantly correlated with increased survival after diagnosis of metastasis. One single variant of *ERBB2* and *AR* and three *MUC16* variants were observed to increase the survival after diagnosis of metastasis highly significantly

The number of likely pathogenic or pathogenic variants was significantly correlated with decreased survival time after diagnosis of metastasis (threshold identified by ROC analysis and Youden’s index: 3.5; *p* value of log-rank test for Kaplan–Meier curve: 0.026; Fig. 4a and Online Resource 6).

Of all tested individual variants with a prevalence between 15 and 85% (*n* = 186), 16 variants (if the variants annotated to the different isoforms are counted separately) displayed a significant correlation [*p* value < 0.05 by log-rank (Mantel–Cox) test] with survival after diagnosis of metastasis (Online Resource 6). The presence of the *BRCA1* c.787 + 1174delA/K607fs*47/K607fs*74 variant (annotated to different *BRCA1* isoforms) significantly correlated with worse survival after diagnosis of metastasis (Fig. 4a), while one *AR* variant, three *MUC16* variants, and one *ERBB2* variant were significantly associated with increased survival after diagnosis of metastasis (Fig. 4b). In addition, the correlation of specific variants with the interval between blood draw and death and the progression-free survival under the therapy given at/after the blood draw was evaluated in all variants with a prevalence between 15 and 85% (*n* = 186; Online Resource 6).

### Longitudinal monitoring across treatment

Here, we describe the development of pathogenic and likely pathogenic cfDNA variants and their AFs in one MBC patient with ER+ PR**–** HER2**–** primary tumor at six time points in a period of 3 years. She was diagnosed with multicentric ductal BC at grade two and osseous metastases in March 2014 at the age of 61 years and died with cutaneous, pulmonary, osseous, pleural, and hepatic metastases in October 2017.

**Fig. 5:**
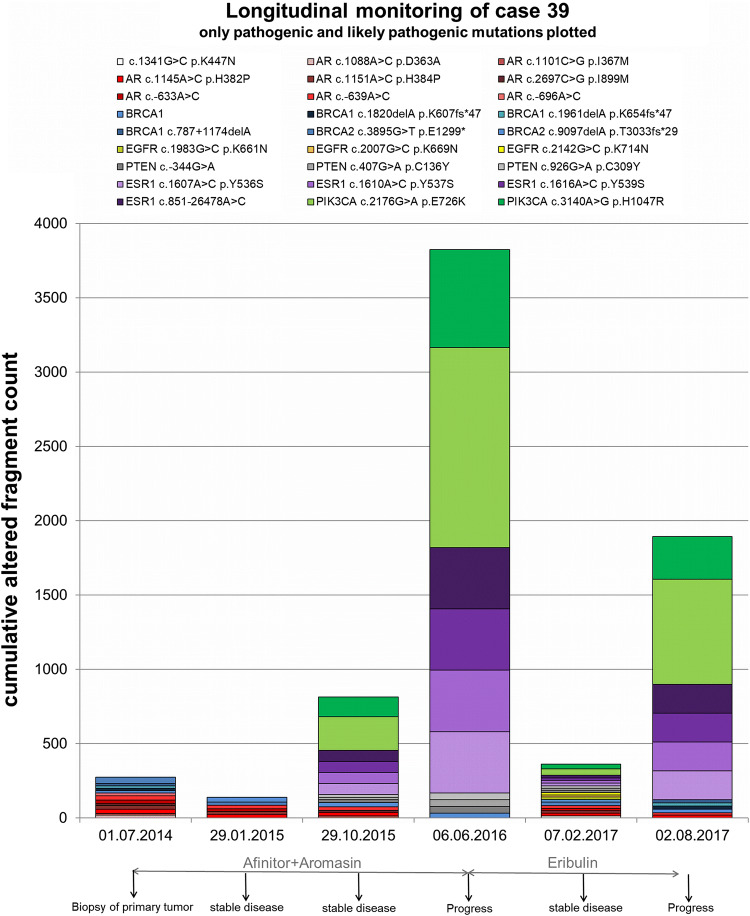
**Longitudinal monitoring of cfDNA variants with pathogenic/likely pathogenic impact in one HR+ HER2**– patient. CfDNA was isolated and sequenced at six time points across treatment in a period of 3 years. Cumulative altered fragment counts of all detected pathogenic and likely pathogenic variants are illustrated. As not to sum allele frequencies, cumulative altered fragment counts are displayed. In general, however, it should be considered that wildtype fragment counts were stable across time points and variant locations, while altered fragment counts exhibited dramatic changes. Variants located in the same gene are shown in the same color but different shade. Applied therapies as well as staging results (by visual staging and RECIST) are depicted as well

In July 2014, the primary tumor was biopsied and the first plasma sample was taken, showing no pathogenic or likely pathogenic variants in the *PIK3CA* or *ESR1* gene (Fig. 5). Afterwards, the patient received everolimus and exemestane which resulted in a stable disease for nearly 2 years until disease progression in June 2016. However, the cfDNA variant development assessed retrospectively in this study showed the appearance and even increase in AF of *PIK3CA* E726K (Oct 2015 9.8%; June 2016 38.3%), *PIK3CA* H1047R (Oct 2015 11.4%; June 2016 39.0%), *ESR1* Y536S, *ESR1* Y537S, *ESR1* 539S and one *ESR1* intronic variant (AF of all *ESR1* variants: Oct 2015 4.1%; June 2016 14.0%) in this time frame. Hence, we identified these pathogenic *PIK3CA* and *ESR1* variants under mTOR and aromatase inhibitor therapy at the staging time point, 8 months earlier than the visual staging time point evaluating a progressive disease. Treatment was then changed to eribulin for about a year until disease progression. Therapy change first induced decreased *PIK3CA* and *ESR1* variant AFs. At the progressive time point under eribulin, however, the *PIK3CA* and *ESR1* variant AFs were dramatically increased again (*PIK3CA* E726K 2.9%–24.6% from Feb 2017 to Aug 2017; *PIK3CA* H1047R 3.3%–26.0% and all *ESR1* variants 1.3%–9.1%). All in all, two *PIK3CA* variants and four *ESR1* variants appeared during everolimus and exemestane therapy and were prominent at both progressive time points within the observed period with > 9% AF.

## Discussion

Deep sequencing of variants in all coding regions of *MUC16*, *BRCA2*, *ERBB3*, *AR*, *EGFR*, *PTEN*, *ERBB2*, *PIK3CA*, *BRCA1*, *ESR1*, *PTGFR*, *TGFB1*, *AKT1*, *FGFR1*, *ERCC4*, *PIK3R1,* and *KRAS* in cfDNA samples of 40 HR+ HER2**–** MBC patients revealed that each patient displayed at least one pathogenic or likely pathogenic variant and the highest frequency of likely pathogenic or pathogenic alterations occurred in the *AR* gene. The prevalence of *MUC16*, *BRCA2*, *ERBB3,* and *AR* variants was > 90% in this cohort. We here identified single variants with significant correlation with increased survival after diagnosis of metastasis. On the other hand, a *BRCA1* variant as well as a number of pathogenic or likely pathogenic variants of greater 3.5 were significantly associated with worse survival after diagnosis of metastasis. The longitudinal monitoring of cfDNA variant evolution of one HR+ HER2**–** patient revealed the prominence of *PIK3CA* and *ESR1* variants before staging the disease to be progressive under everolimus and exemestane.

### Implications of input amount, UMI integration, high coverage, and IVA filter

The required blood volume of 9 ml is comparable to the obligatory blood volume for other liquid biopsy tests used in clinical practice already (such as Guardant360^®^ ctDNA [19] or OncoBEAM™-RAS-Test) yielding in minimally 4 ml plasma. The high plasma input of 4 ml enabled isolation of cfDNA amounts usable for reliable targeted PCR-based cfDNA library preparation (library yield > 4 nM). The integration of UMIs was exemplified for identification of *PIK3CA* hotspot variants in this study and guarantees the identification of only true positive variants, but requires high cfDNA input and deep sequencing qualities. Exactly those deep sequencing qualities were met in all 40 cases used for variant analysis, since 18 million read fragments were sequenced on average in each sample, the mean coverage was 22,000×, and the UMI coverage was 6351. This high coverage enabled calling variants with low AFs and resulted in the identification of a high prevalence of variants, which has rarely been described before [20–22]. Many studies [2, 23–25] that described a low frequency of variants were conducted with a lack of high coverage, missing descriptions of the input amount or sequencing of hotspots rather than all exonic regions of a gene. We here ensure specificity and relevance of called variants by usage of UMIs and stringent IVA filter settings [16] and variant prevalence detected by analysis of tissue or liquid biopsy should be differentiated. Among other settings, only predicted deleterious variants were called and variants with a prevalence of > 3% in the healthy reference population (Allele Frequency Community (gnomAD&CGI), 1000 Genomes Project, ExAC and NHLBI ESP exomes) were excluded. Classification of the variants was conducted according to the guidelines of the association for molecular pathology [26]. Although efforts ensuring a high sensitivity and specificity in variant calling were undertaken, a verification of variants by independent methods in the future would be desirable, which was not conducted here due to the limited cfDNA amount available and the plenty of variant locations to be verified. The detected sequence alterations were separately listed with different variant nomenclature when different isoforms were concerned. This can be one reason for the large number of reported variants. Although we did not compare the data against cfDNA of healthy donors that underwent the same workflow, we excluded variants with a high prevalence in the reference populations. Matched germline samples were not available, thus, we do not claim the described variants to be somatic per se, but low AFs might indicate the variants to be somatic rather than germline and sequencing of matched germline samples has been initiated for future studies.

### *MUC16* variants

*MUC16* variants were reported to be frequently found in most cancer types [27, 28] which is in line with the high prevalence of *MUC16* variants in our patient cohort. However, due to the fact that *MUC16* consists of ~ 22,000 amino acids, the variant frequency corrected by sequence length and mutational heterogeneity resulted in exclusion of *MUC16* as one of the most altered genes in most cancer types, including BC [28, 29]. *MUC16* variants were found to induce MUC16 overexpression in BC and thus caused increased cancer growth and migration as well as decreased sensitivity to cisplatin [30]. The presented new highly significant association of three *MUC16* variants (R10996S, S836R, P835A) with increased survival after diagnosis of metastasis challenges the knowledge about the negative effect of *MUC16* variants in BC. In accordance with the statement of decreased sensitivity to cisplatin [30], preliminary data of our cohort showed that the mean number of *MUC16* variants was reduced in the cohort that had received platinum-based therapy sometime before blood draw (*n* = 4) compared to those patients who had never received any platinum-based therapy (*n* = 34) (15 versus 28 *MUC16* variants), but no significant reduction in survival time comparing both cohorts (107 months versus 117 months) was found (data not shown). A cluster analysis of all *MUC16* variants further separated the patients into two distinct groups, but these data are too preliminary to draw any clinical conclusions.

### Nuclear steroid hormone receptor variants

*ESR1* variants were mostly found in ER+ patients and were induced by endocrine treatment [2, 31]. *ESR1* variants in cfDNA of MBC patients were reported to have a prevalence of 25.3% (PALOMA3), 28.8% (BOLERO-2) and 39.1% (SoFEA) [6]. The 55% *ESR1* variant prevalence in the presented study may be explained by the inclusion of a majority of HR+ cases in late treatment lines. In this regard, it is to mention that 62% of all included patients received two or more different endocrine treatment regimens before blood draw including non-steroid aromatase inhibitors, steroid aromatase inhibitors, selective estrogen receptor modulators, or selective estrogen receptor degraders (Online Resource 1).

Longitudinal cfDNA monitoring in one of the patients identified the appearance and increase in AF of *ESR1* Y536S, *ESR1* Y537S, and *ESR1* Y539S which might be one potential mechanism of resistance causing progressive disease after 2 years of endocrine treatment. These three variants are located at positions encoding for the ligand-binding domain of ER and cause resistance to aromatase inhibitor therapy and, thus, were already described to be predictive [2, 8, 32–34].

AR expression on the mRNA- and protein level was studied in BC cases after realization of a complex interplay of androgens and estrogens [35]. However, to the best of our knowledge, we are one of the first studying the genomic alterations in the *AR* gene in BC in more detail and strikingly found that 88% of all patients displayed likely pathogenic and pathogenic (defined by guidelines of the association for molecular pathology [26]) *AR* variants. Almost half of all likely pathogenic and likely pathogenic variants found in all tested 17 genes were localized in the *AR* gene, as previously described [18]. Such high prevalence of *AR* variants should be further studied to elucidate the functional influence of *AR* variants on the AR expression, subsequently, on AR-dependent pathways and, finally, on BC pathogenesis. DNA regions encoding for glutamine repeats, also called polyQ stretches, are frequently found in the *AR* gene [36] and were described to be of high functional relevance [36]. However, variant calling in polyQ stretches was found to be challenging [37]. *AR* splice variants were already detected in BC patients [38] and *AR***-***V7* was reported to cause resistance to anti-AR therapies in prostate cancer [39]. Consequently, the functional analysis of *AR* variants might indicate new treatment options for BC patients with *AR* variants in the future.

### *PIK3CA* variants

*PIK3CA* variants showed a high prevalence in HR+ HER2**–** BCs (45%/29% of luminal A/B BC patients) [40]. Here, we detected *PIK3CA* variants in 73% of HR + HER2-BC cases. This increased prevalence may be due to the high number of patients included who had received more than three treatment lines, since a higher *PIK3CA* variant prevalence was described for heavily pretreated cohorts [41].

*PIK3CA* E726K has already been detected in BC tissue and was predicted to cause a gain of function as well as increased oncogenic transformation, which was also described for *PIK3CA* H1047R located in the activation loop of the kinase domain [42–44]. With regard to conferred mechanisms of resistance, sensitivity to everolimus was not affected by *PIK3CA* variants [45]. However, *PIK3CA* variants affected the PIK3/AKT pathway activation and the latter caused endocrine resistance [46]. Thus, besides the detected *ESR*1 variants, the here described variants *PIK3CA* E726K and *PIK3CA* H1047R with dramatically increased AF under everolimus and exemestane in the serial cfDNA samples of one patient might mirror another resistance mechanism to exemestane.

### Variants of the ErbB protein family

We found high prevalence of variants in the genes of the three ErbB protein family members *ERBB2*, *ERBB3,* and *EGFR.* This underlines the importance of *EGFR* for BC growth and progression in HER2**–** BC cases [47]. Interestingly, however, *ERBB2* P582L, *ERBB2* P597L, and *ERBB2* P612L, all causing the change of proline to leucine, were found to be significantly associated with increased survival after diagnosis of metastasis. This finding questions the clinical conclusion of the genomic variants found in plasma, since the increased survival observed in *ERBB2* altered cases might be due to effective targeting of *ERBB2* altered tumor cells by therapy to result in detection of these genomic variants in the plasma [48].

### Variants of the BRCA protein family

Alterations in proteins involved in the DNA repair pathways have a high impact on carcinogenesis. Individuals with germline *BRCA2* variants have an incidence of 57%–65% to develop BC [49]. BC patients with *BRCA1* or *BRCA2* mutations, however, were demonstrated to be sensitive to platinum agents and poly (ADP-ribose) polymerase (PARP) inhibitors [50]. Using deep sequencing of cfDNA, we here described a prevalence of *BRCA1/2* variants in 95%/60% of the cohort. This high prevalence of *BRCA* variants in cfDNA of HR+ HER2**–** MBC patients has never been described before, probably also due to limitations of coverage capacities [51–53]. It is of note that the mean AF of *BRCA1/2* variants found in the cohort was 1.5%/3.9%, indicating the detection of somatic rather than germline variants.

### Prognostic value of cfDNA characteristics

The accumulation of likely pathogenic and pathogenic variants might cause survival disadvantages for the patients, since we found a significant correlation of the number of pathogenic and likely pathogenic variants with survival after diagnosis of metastasis.

### Longitudinal monitoring across treatment

The longitudinal cfDNA variant monitoring in one patient does not mirror the entire cohort, but was chosen as an example for the evolution of variants in the course of the disease, which might also be taken as an indicator of certain selective pressure that seems to happen during the observation time. The high AFs of *PIK3CA* and *ESR1* variants at progression under eribulin are consistent with the knowledge about the persistence of aromatase inhibitor-selected *ESR1* variants throughout subsequent therapies [2] and a general increase of variant AFs at therapy failure [54]. Due to the fact that all variants detected at the time point of progression under eribulin had already been detected at the previous sampling time points, the mechanism for acquired resistance to eribulin might not be caused by the observed variants themselves.

Interestingly, the appearance of *ESR1* and *PIK3CA* variants with AFs > 4% was already observed 8 months earlier than progression of the disease by visual staging. Thus, these variants appear more sensitive in monitoring the evolution and potential resistance than contemporary staging methods. The promising effect of cfDNA monitoring, in general, to predate treatment response changes by weeks to months was described before [11, 12].

In conclusion, comprehensive analysis of cfDNA variants by targeted deep sequencing in HR+ HER2**–** BC cases not only confirmed the benefit of UMIs in the variant verification, but also identified new promising variants with clinical relevance for monitoring, prognosis, and therapy stratification. More comparative research needs to be done, focusing on other BC subtypes and also on other BC relevant genes to further emphasize the advantages of deep sequencing of cfDNA for BC therapy management.

### Electronic supplementary material

Below is the link to the electronic supplementary material.

Supplementary material 1 (XLSX 15 kb)

Supplementary material 2 (XLSX 17 kb)

Supplementary material 3 (XLSX 14 kb)

Supplementary material 4 (XLSX 13 kb)

Supplementary material 5 (XLSX 29 kb)

Supplementary material 6 (XLSX 12 kb)

## Abbreviations

AF: Allele frequency
BC: Breast cancer
cfDNA: Cell-free DNA
ctDNA: Cell-free tumor DNA
ER: Estrogen receptor
HR: Hormone receptor
IQR: Interquartile range
IVA: Ingenuity variant analysis
NGS: Next generation sequencing
MBC: Metastatic breast cancer
PR: Progesterone receptor
UMI: Unique molecular indice

## List of genes

*AKT1*: *AKT serine/threonine kinase 1*
*AR*: *Androgen receptor*
*BRCA1*: *BRCA1 DNA repair associated*
*BRCA2*: *BRCA2 DNA repair associated*
*EGFR*: *Epidermal growth factor receptor*
*ERCC4*: *ERCC excision repair 4, endonuclease **catalytic subunit*
*ERBB2*: *Erb*-*b2 receptor tyrosine kinase 2: encoding for HER2*
*ERBB3*: *Erb*-*b2 receptor tyrosine kinase 3: encoding for HER3*
*ESR1*: *Estrogen receptor 1*
*FGFR1*: *Fibroblast growth factor receptor 1*
*KRAS*: *KRAS proto*-*oncogene, GTPase*
*MUC16*: *Mucin 16, cell-surface associated*
*PIK3CA*: *Phosphatidylinositol*-*4,5*-*bisphosphate 3*-*kinase catalytic subunit alpha*
*PIK3R1*: *Phosphoinositide*-*3*-*kinase regulatory subunit 1*
*PTEN*: *Phosphatase and tensin homolog*
*PTGFR*: *Prostaglandin F receptor*
*TGFB1*: *Transforming growth factor beta 1*
